# Investigate the efficiency of primary care facilities in emergency situations by application of geographical and demographic standards using GIS

**DOI:** 10.12688/f1000research.140107.2

**Published:** 2024-07-05

**Authors:** Ali E. M. Jarghon, Nyoman Anita Damayanti, Inge Dhamanti, Anas M. M. Awad

**Affiliations:** 1 Faculty of Public Health, Airlangga University, Surabaya, East Java, 60114, Indonesia; 2 Faculty of Geodesy and Geomatics Engineering, Bandung Institute of Technology, Bandung, West Java, 40132, Indonesia

**Keywords:** Primary health care, emergencies, geographical standard, demographic standard, GIS, Coverage

## Abstract

**Background:**

This study applied geographical standards (coverage distance) and demographic standards to investigate the capabilities of primary healthcare facilities to cover the largest area of the study area and the service area that can be accessed. This study was conducted to find out the sufficient number of primary healthcare (PHC) centers required to provide healthcare services to the entire community.

**Methods:**

Data was obtained by applying geographic information system (GIS) techniques to analyze primary care facilities using the demographic and geographic standards for primary care facilities.

**Results:**

PHC centers cover 79% of the study area according to the geographical standard. The study area needs 41 additional centers to cover the shortfall in service provision per the demographic and geographic standards.

**Conclusions:**

A significant deficiency in the number of primary care centers found in the study area compared to the large population at the geographical and demographic standards level.

## Introduction

The pre-disaster phase of the disaster management (DM) process is based on examining the current readiness of facilities and services related to disaster risk reduction and identifying and addressing deficiencies and weaknesses for providing the needs and requirements for the success of the DM process. ^1^ The spatial evaluation process for the sites of services and facilities needed for DM examines readiness in the study area using geographic information system (GIS) techniques and spatial analysis. ^2^^–^^4^

Geographical standards (coverage distance) and demographic standards show the capabilities of primary health center facilities to cover the largest area of the study area and the service area. In addition, we analyze the population’s suitability with the number of facilities available in the study area to find out if it is sufficient to provide services to the entire community according to the standards required. ^5^^–^^8^

### Planning standards for primary care centers

Primary health care (PHC) is a crucial component of health systems and communities’ social and economic development. ^9^^–^^11^ PHC is responsible for providing health services and responding to emergencies at each sub-region level. ^11^ There are 26 centers in Sleman District, Indonesia, distributed over 17 sub-regions. ^12^

The study utilized demographic and geographic Standards as variables, gathering data from the District Health Office. It adhered to the Ministry of Health’s regulations to assess their applicability within the study area.

## Methods

### Study area

The study was conducted in Sleman District, Yogyakarta City, Indonesia, which comprises 26 Primary Health Care (PHC) centers distributed across 17 sub-regions. Sleman District has the highest disaster risk index in Yogyakarta, with a score of 97. ^19^ This study utilized ArcGIS 10.5 to analyze the distribution and accessibility of PHC facilities based on demographic and geographic standards, aiming to identify shortages, assess needs, and propose solutions and recommendations. ^20^^,^^21^^,^^27^

The GIS-based analytical approaches employed in this study depend on the availability of spatial and non-spatial data. ^28^ According to the geographical standard, each sub-district should have one PHC, and urban facilities should have a service scope within specific distances: 5 km for hospitals, 2.5 km for schools, and 2 km for markets. Non-spatial data, such as population demographics, were also considered to estimate healthcare demand. ^17^^,^^22^

Furthermore, the study utilized statistical data to measure the need for new PHC centers based on the population data. The Ministry of Health set the standard where each PHC has to provide services for 20,000 people. ^18^

## Data collection and preparation

### Astronomical location

**Figure 1.**
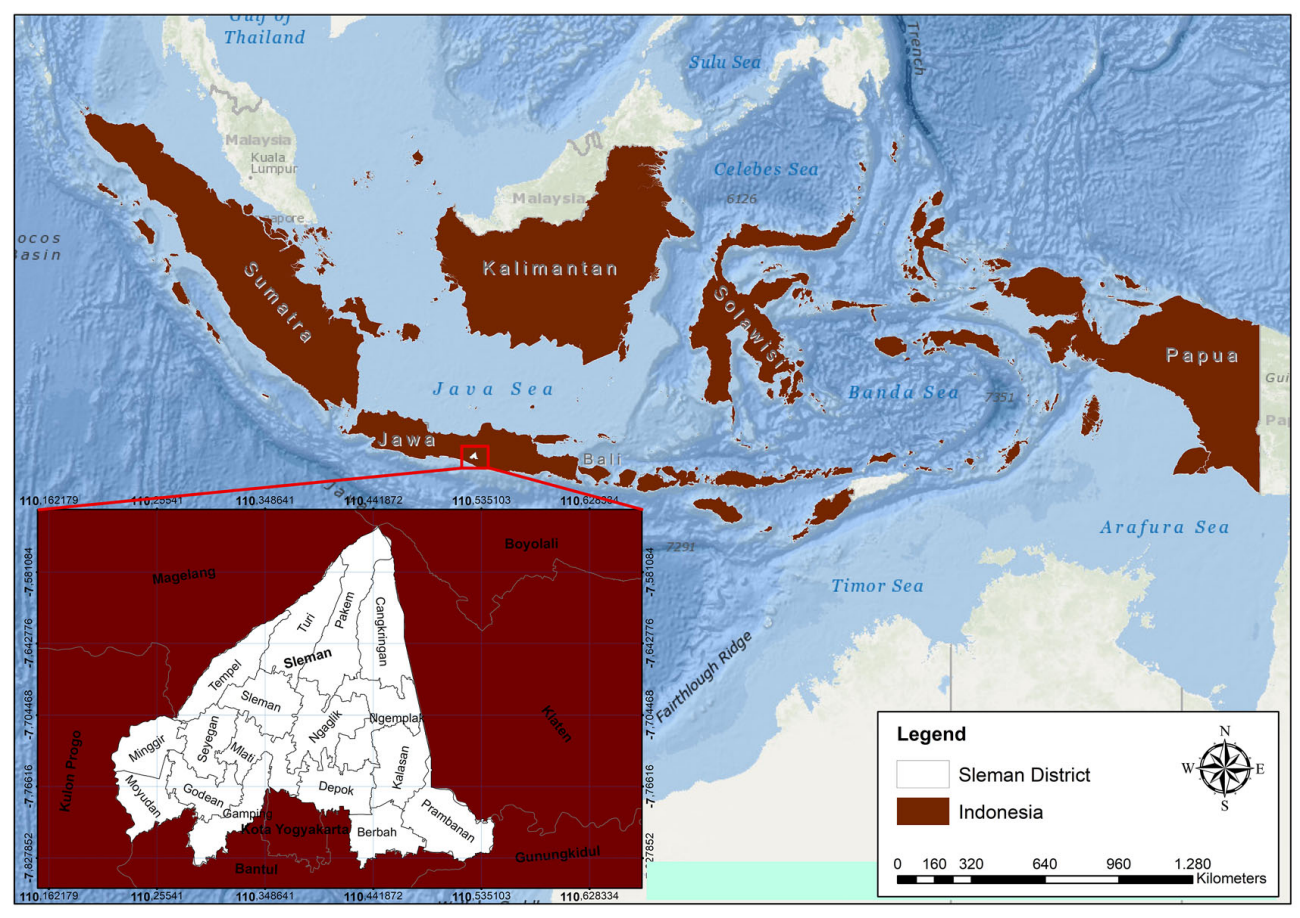
Astronomical site of Sleman district.

Sleman District is located between 110° 13′ 00″ to 110° 33′ 00″ east longitude and 7° 34′ 51″ to 7° 47′ 03″ south latitude (see Figure 1).

### Population

Sleman had a population of 1,206,714, comprising 608,968 males and 597,746 females, within an area of 574.82 km ^2^. The population density was 2,099 people per km ^2^. Notably dense districts include Depok (5,359 people per km ^2^), Mlati (4,049 people per km ^2^), Gamping (3,771 people per km ^2^), and Ngaglik (3,194 people per km ^2^) in 2018. ^23^

### Data collection, processing, and analysis

Proximity Analysis: Assessed the accessibility of PHC facilities by measuring distances or travel times from specific locations, considering transportation networks and road conditions to determine ease of access during emergencies.

Spatial Overlay: Combined spatial data layers to identify relationships and patterns, such as overlaying facility locations with demographic data to pinpoint areas with a critical need for primary care.

Network Analysis: Optimized routes and travel times using transportation networks, aiding in the identification of efficient emergency service routes and areas requiring improved infrastructure or additional facilities. ^29^^,^^30^

**Figure 2.**
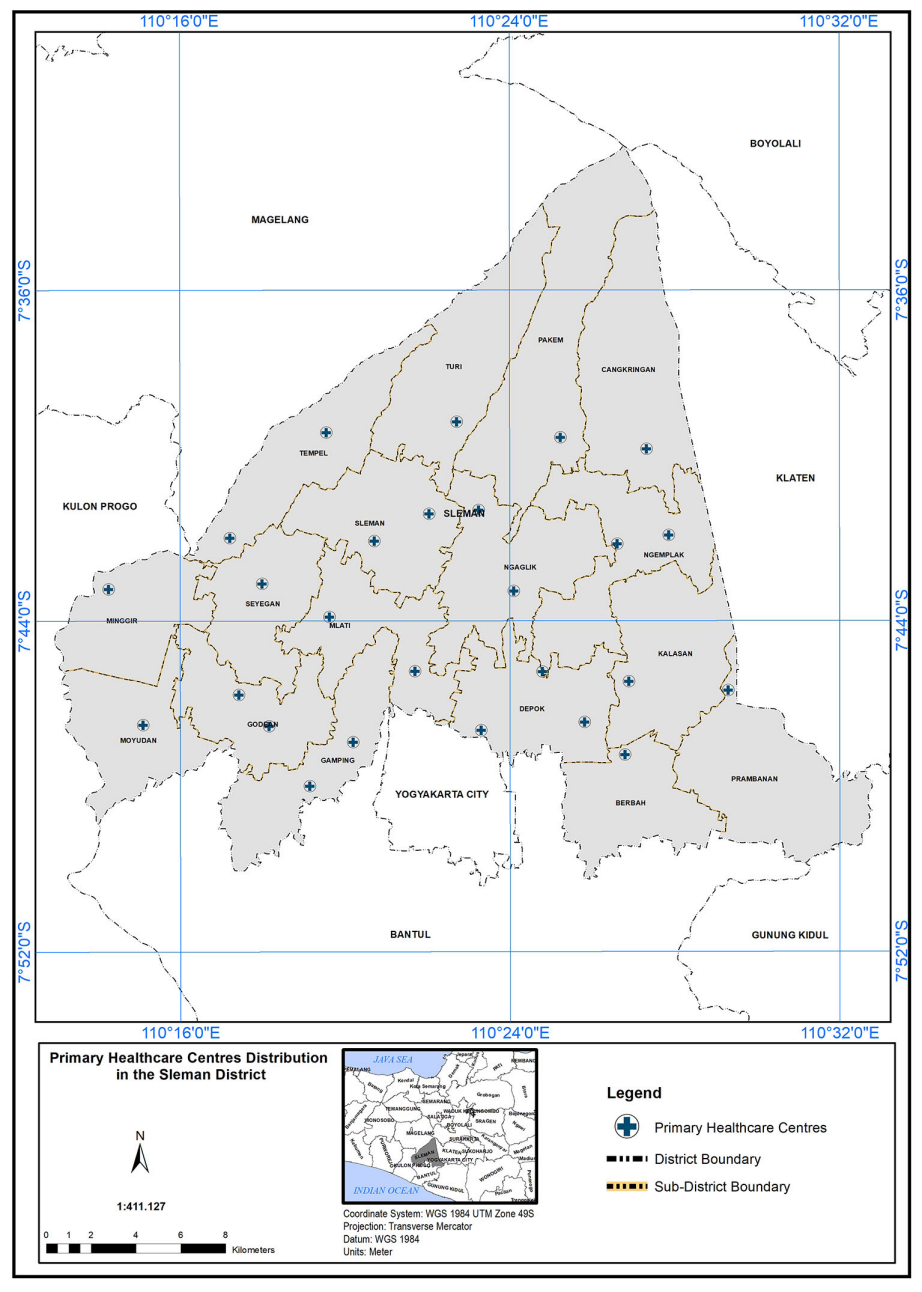
Primary healthcare center distribution in Sleman district.

The correctness of facilities’ locations were verified through maps using Google Earth and WorldWindEarthExplorer. Using the ArcGIS 10.5 program and Arc Analysis tools, the study area is derived based on arial images, identified by comparing it with the arial images obtained from Indonesian Disaster Management Agency. The extracted data sets were then transferred to Microsoft Excel 2013, using the network analysis in Arc GIS 10.5 (open source alternative: QGIS) to analyze and determine the scope of service of PHC. Spatial analysis of the location regarding PHC needed in the study area depended on geographical standard and demographic standards. By applying these standards, we can inquire about PHC capabilities to cover the study field and the service area that could be accessed. In addition to analyzing the population’s suitability with the number of PHCs available in the study area, is it sufficient to provide primary healthcare services to all, at the time and standard required? The analysis started by obtaining the map of 26 PHC centres distributed over 17 sub-regions as shown in Figure 2.

## Results

### Geographical standard (coverage distance)

According to the geographical standard, each sub-district must have one PHC and urban facilities, including a scope of service within 5 km, a school within 2.5 km, a market within 2 km, and a hospital within 5 km. ^25^ The service area of PHCs in the study area, based on these geographical standards, is illustrated below:

### Geographical standard, availability of hospital within 5 km

**Figure 3.**
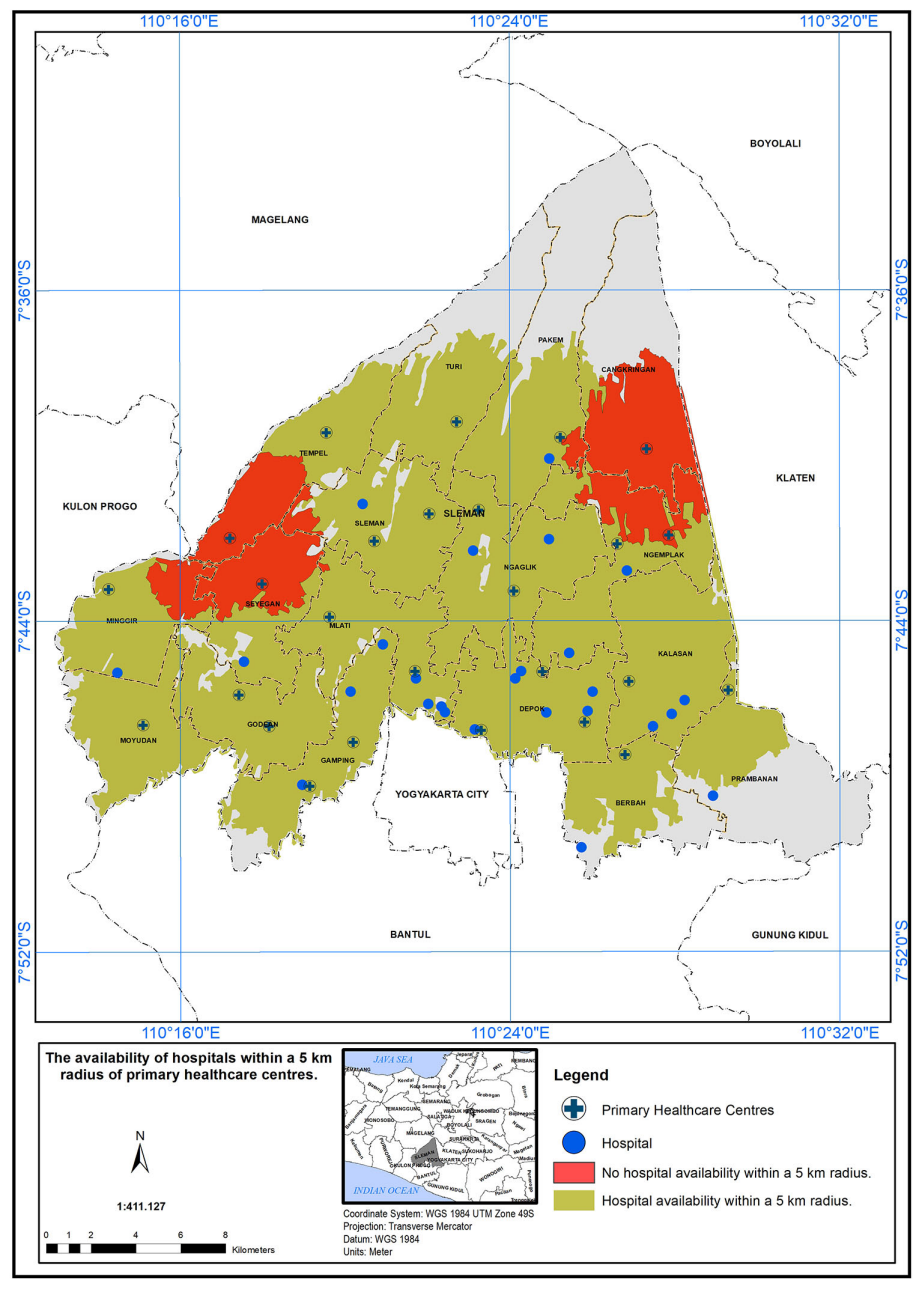
Illustrates the service area of primary healthcare (PHC) in the study area, based on a geographical standard of 5 km from the hospitals.

The PHC service areas in the study area are defined by a 5 km radius from hospitals. Figure 3 shows the availability of hospitals within this radius. It was found that 24 health centers met the specified standard in the presence of a hospital (represented in green), while two health centers did not meet the standard (represented in red).

### The scope of service within 5 km

**Figure 4.**
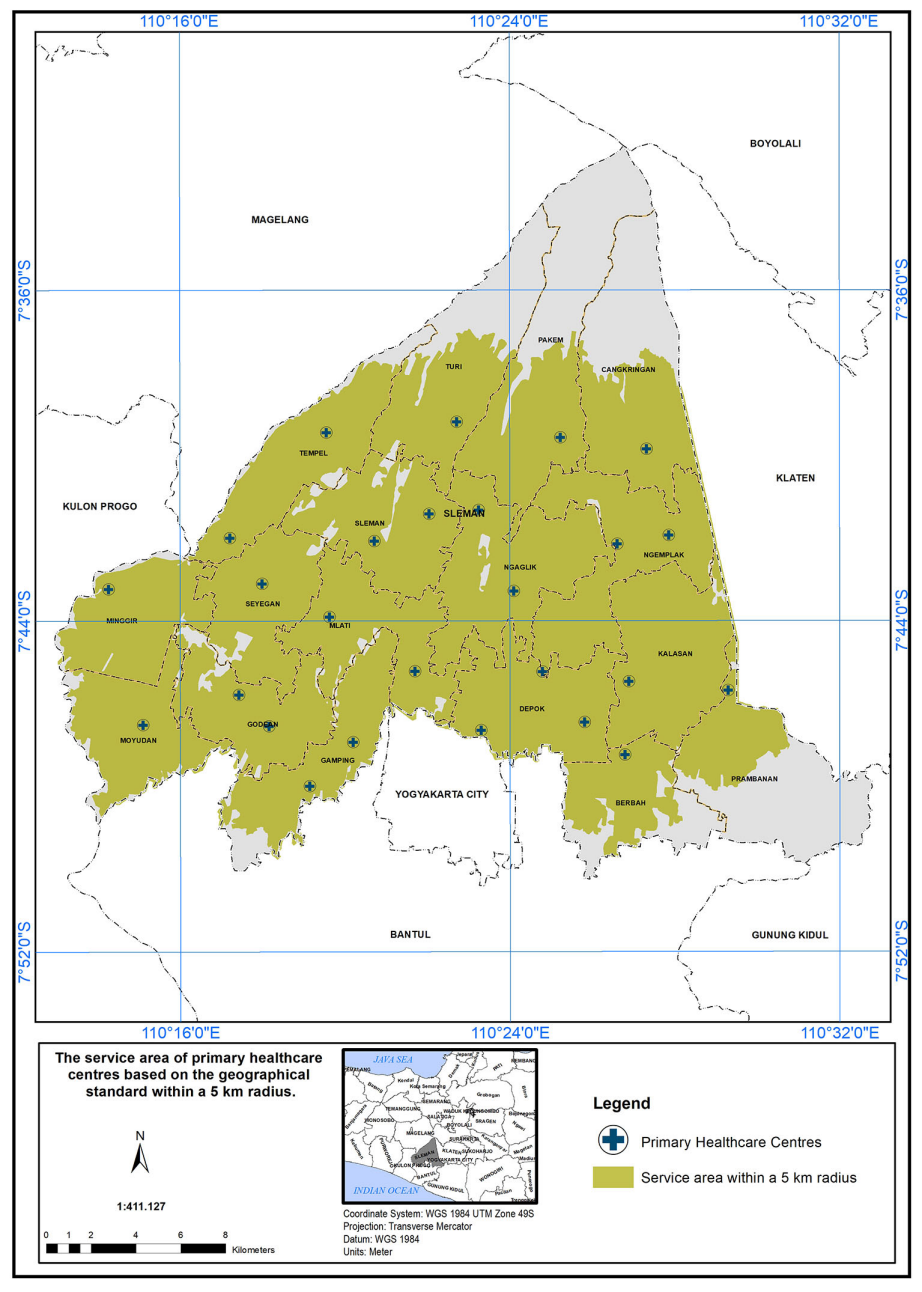
The service area of PHC in the study area based on the geographical standard (5 km).

The PHC service area based on a 5 km radius is further detailed in Figure 4. According to the Indonesian Ministry of Health standards, health centers are required to provide services within a 5 km distance. In Sleman District, health centers cover an area of 454.10 km ^2^ out of 574.25 km ^2^, serving approximately 79% of the study area. The unserved area totals 120.15 km ^2^, primarily in the northern (Cangkringan, Pakem, Turi, Tempel) and southern (Prambanan, Berbah, Gamping) districts. Detailed coverage for each district is provided below:

• Cangkringan District: One PHC covers 24.24 km ^2^ out of 44.72 km ^2^ (45% unserved).
• Pakem District: One PHC covers 25.98 km ^2^ out of 52.70 km ^2^ (50.7% unserved).
• Turi District: One PHC covers 22.90 km ^2^ out of 40.50 km ^2^ (43.4% unserved).
• Tempel District: Two PHCs cover 28.45 km ^2^ out of 32.33 km ^2^ (12% unserved).
• Prambanan District: One PHC covers 14.25 km ^2^ out of 40.62 km ^2^ (64.9% unserved).
• Berbah District: One PHC covers 17.0 km ^2^ out of 25.85 km ^2^ (24.2% unserved).

In conclusion, the study area has a service coverage deficiency of 21%. This highlights the need for additional primary healthcare centers (PHCs) in districts that face shortages. These districts have high population densities and are also located in close proximity to natural disaster sources, such as volcanoes, floods, and landslides.

### Geographical standards, availability of schools with 2.5 km

**Figure 5.**
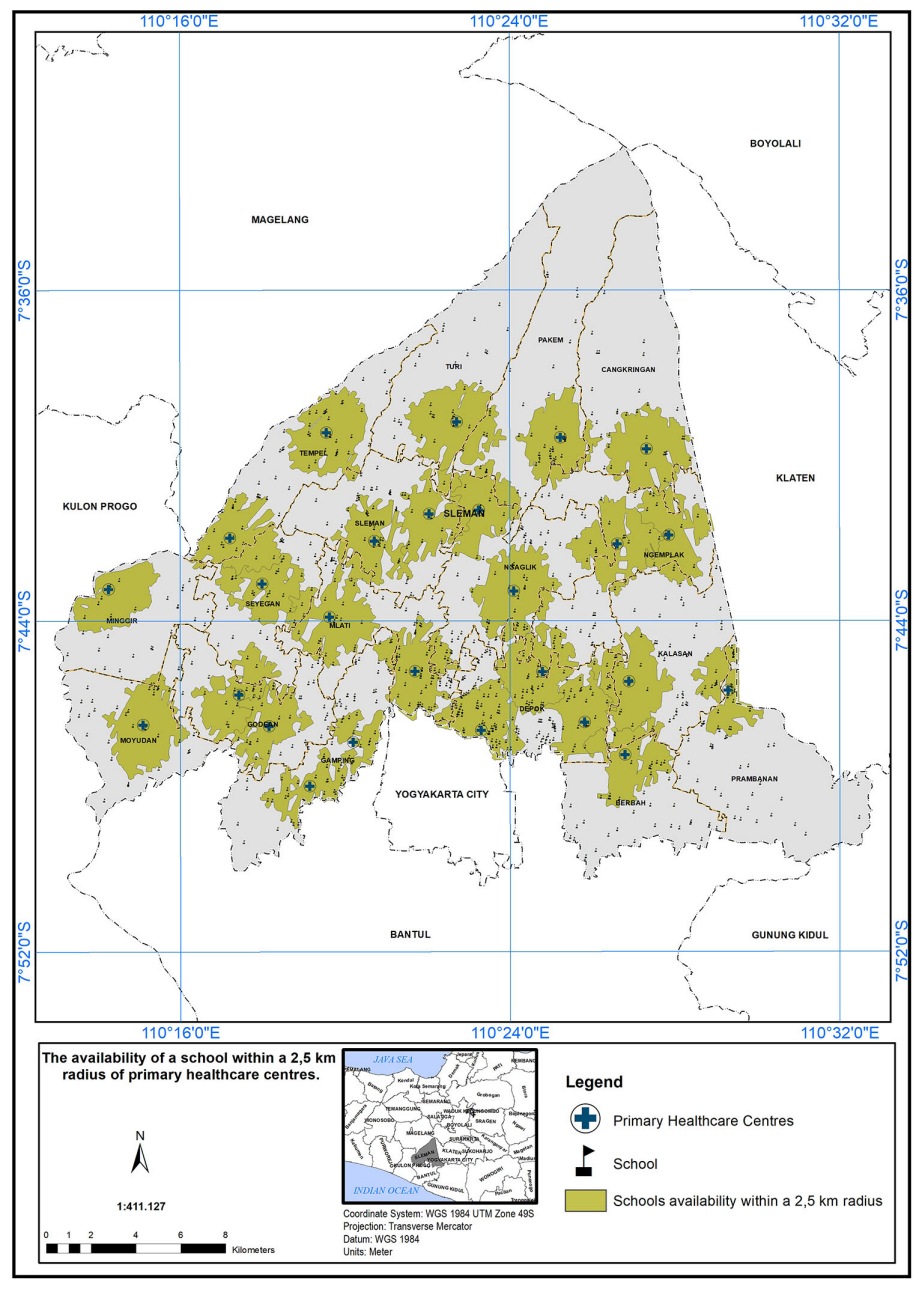
The service area of the PHC centre (2.5 km from the schools).

The service area of the PHC center is defined as a 2.5 km radius from the schools. Figure 5 illustrates the availability of schools within this 2.5 km radius. A service scope within 2.5 km of the primary care centers has been implemented. In Figure 5, 26 PHC centers met the specified standard in the presence of a school, indicated by the green color.

### Geographical standards, availability of market within 2 km

**Figure 6.**
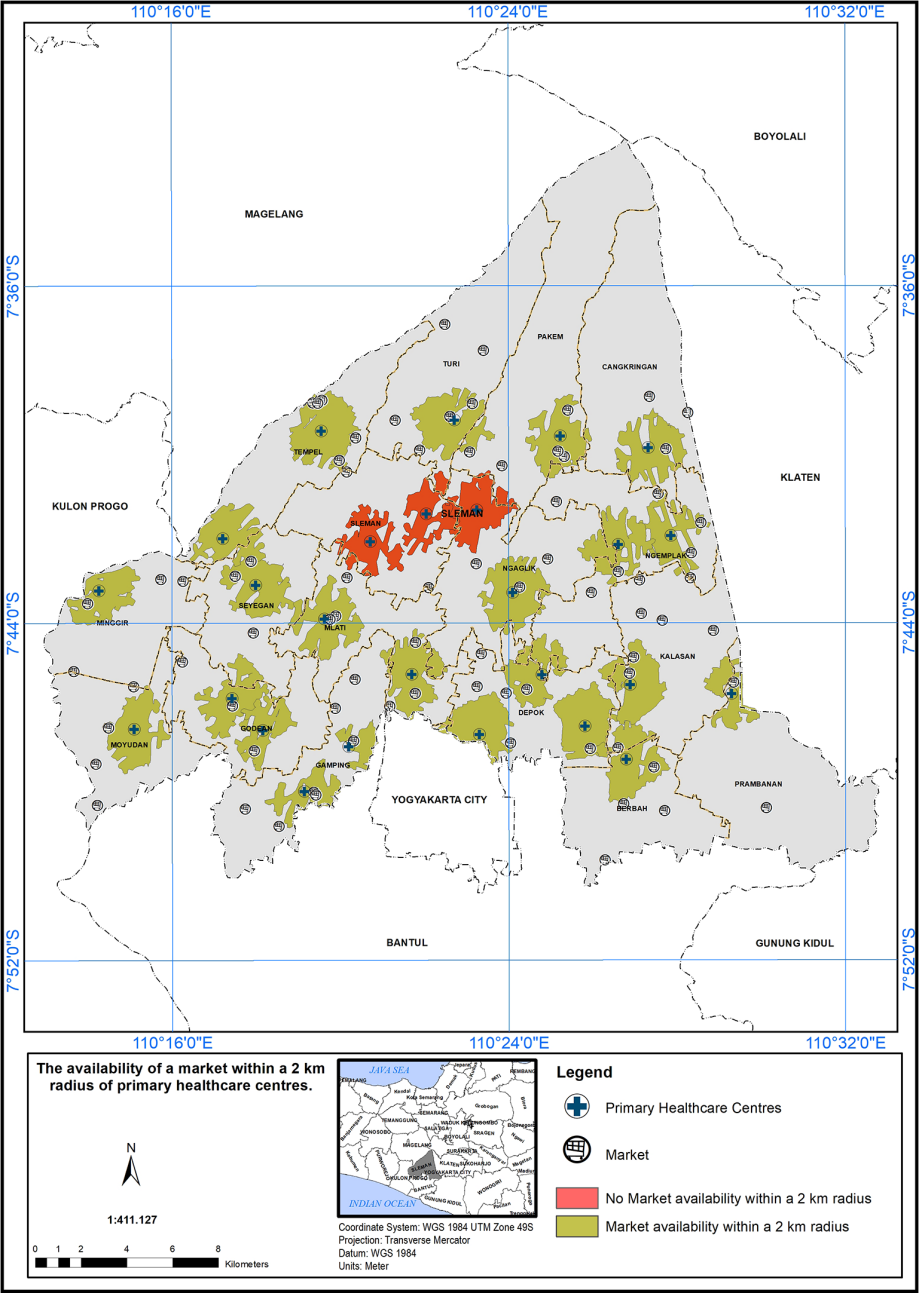
The service area of the PHC centre in the study area based on the geographical standard (2 km from the market).

The service area based on a 2 km radius from markets is shown in Figure 6. Out of 26 PHCs, 23 met the standard (indicated in green), while three did not (indicated in red). Three PHC centers did not meet the specified standard in the presence of a market, indicated by the red color.

### Demographic standard

Table 1 provides an explanation of the individuals who receive services and those who do not receive services through the PHC center in Sleman District, based on the demographic standard. The location of the PHC center is determined by the population distribution in the region, following the demographic standard set by the Indonesian Ministry of Health, which specifies serving a population of 20,000 people per PHC. ^24^ After applying the demographic standards, the study yielded the following findings. The total population of the Sleman district is 1,206,714 people. Out of 26 PHCs, 520,000 individuals are receiving services in accordance with the prescribed criteria. The population without access to services amounts to approximately 686,714 people. According to the demographic standard, a larger percentage of the population should have access to PHC services. The shortage in health centers represents approximately 41 PHC centers. Statistical data indicates that the highest number of individuals lacking services in PHCs is found in the Depok region, with a shortage of 130,526 people. The construction of seven additional PHC facilities is necessary. In contrast, the percentage of individuals lacking access to PHC services in Cangkringan District is relatively small, necessitating the establishment of one new PHC.

**Table 1.** Number of people who receive services and who do not receive services through PHC centre according to the demographic standard in Sleman District.

No.	Sub-District	Population (2018)	No. of PHCs	Served population	Unserved population	PHCs needed
1	Berbah	59,943	1	20,000	39,943	2
2	Cankringan	29,592	1	20,000	9,952	1
3	Depok	190,526	3	60,000	130,526	6-7
4	Gamping	110,288	2	40,000	70,288	3-4
5	Dodean	72,286	2	40,000	32,286	1-2
6	Kalasan	88,110	1	20,000	68,110	3-4
7	Minggir	29,929	1	20,000	9929	1
8	Mlati	115,466	2	40,000	75,466	3-4
9	Moyudan	31,536	1	20,000	11,536	1
10	Ngaglik	123,039	2	40,000	83,039	4
11	Ngemplak	66,899	2	40,000	26,899	1-2
12	Pakem	38,658	1	20,000	18,658	1
13	Prambanan	48,734	1	20,000	28,734	1-2
14	Seyegan	47,355	1	20,000	27,355	1-2
15	Sleman	68,480	2	40,000	28,480	1-2
16	Tempel	50,844	2	40,000	10,844	1
17	Turi	34,489	1	20,000	14,489	1
Total	17	1,206,714	26	520,000	686,714	

## Discussion

In this study, the researchers set a geographical standard to determine the service area of Primary Health Care (PHC) centers in the study area. The standard was based on a 5 km radius from the hospitals. ^17^ According to the findings presented in the study, it was observed that 24 health centers met the specified standard for service area coverage in the presence of a hospital. This discrepancy between compliant and non-compliant health centers may have important implications for healthcare accessibility in the region. The availability of PHC services within a 5 km radius from hospitals could significantly impact the ease of access to healthcare for the population living in those areas. The PHC centers that fall within the service area are better positioned to serve their communities effectively, ensuring that a larger population has access to essential healthcare services. Factors such as distance, transportation infrastructure, and population density could play a role in determining the feasibility of establishing PHC centers in those regions. ^26^ Policymakers and healthcare administrators may need to consider additional measures to address the accessibility challenges faced by these areas, potentially by improving transportation options or establishing additional health facilities to fill the service gap.

Overall, this study provides valuable insights into the distribution of PHC services based on geographical standards in the study area. The findings can serve as a foundation for healthcare planning and resource allocation, aiming to enhance healthcare accessibility and ultimately improve the overall health outcomes for the population in the region. However, further research and collaboration between various stakeholders, including government agencies, healthcare providers, and community representatives, will be necessary to address the challenges highlighted by the study and ensure equitable access to healthcare services for all residents.

The variations in PHC coverage across different sub-districts signify the importance of localized and targeted healthcare planning and resource allocation. It is evident that certain areas are more underserved than others, requiring specific attention and efforts to improve healthcare accessibility in those regions.

The study’s findings can serve as a foundation for evidence-based decision-making in healthcare planning and policy development. By identifying the areas with the greatest need for additional healthcare resources, policymakers can prioritize their interventions to achieve more equitable healthcare distribution and improve the health outcomes of the population in the study area. The implementation of a service scope based on a 2.5 km radius from schools is a commendable step towards improving healthcare accessibility and promoting the well-being of the school community.

It also encourages early intervention and preventive healthcare practices, which can contribute to better health outcomes among the school community.

The implementation of a service scope based on a 2 km radius from markets is a promising initiative to enhance healthcare accessibility in the study area. ^17^ The findings in Figure 6 underscore the importance of such approaches in healthcare planning. However, it is crucial to continue evaluating and refining the service area to ensure that healthcare services effectively reach all populations, particularly those residing near markets. By addressing the challenges and disparities identified in the evaluation process, healthcare providers and policymakers can work together to build a more inclusive and accessible healthcare system, ultimately contributing to the improvement of community health and well-being.

The study highlights the importance of adhering to demographic standards to ensure that a larger percentage of the population has access to PHC services. There is a shortage of 41 PHC centers, based on the demographic standard of 20,000 people per PHC. This shortage of health centers directly affects the number of individuals who lack access to healthcare services in the region.

The study’s results have significant implications for healthcare planning and resource allocation in Sleman District. Policymakers and healthcare authorities need to take into account the demographic standards and the actual healthcare needs of the population when planning the distribution and establishment of PHC centers.

The study identifies significant gaps in healthcare access for a substantial portion of the population. Addressing these disparities requires careful planning, resource allocation, and the establishment of new PHC centers, particularly in regions with the greatest need. By addressing the shortages and expanding healthcare infrastructure, policymakers can work towards achieving a more equitable healthcare system that caters to the healthcare needs of all individuals in the district.

## Conclusion

In conclusion, there is a significant deficiency in the number of PHCs in the study area compared to the large population, both at the geographical and demographic standards level. The region is in urgent need of filling these gaps by establishing new PHC centers to meet the population’s needs and provide high-quality PHC services in line with international standards. This will also help alleviate the burden on central hospitals in the region.

## Data Availability

The data for this study are owned by the Indonesian Disaster Management Agency, Statistics Indonesia (BPS), and the Sleman District Health Office, it can be obtained here:
1. Click the following link, change language to English, click download publication, input your E-mail, and the file will be downloaded https://slemankab.bps.go.id/publication/2019/08/16/c400805c8dee98a3d701ea33/kabupaten-sleman-dalam-angka-2019.html
2.
https://geoportal.slemankab.go.id/; to access the data, use the direct link as following: https://geoportal.slemankab.go.id/layers/geonode_data:geonode:a__3404_50KB_PT_SEBARAN_RUMAH_SAKIT_SLEMAN_20 https://geoportal.slemankab.go.id/layers/geonode_data:geonode:a__3404_50KB_PT_PUSKESMAS_SLEMAN_2020
