# Supplementary material for: Investigate the efficiency of primary care facilities in emergency situations by application of geographical and demographic standards using GIS
Source: F1000Res. 2025 Dec 19;12:1579. Originally published 2023 Dec 13. [Version 4] doi: 10.12688/f1000research.140107.4 (PMC11519617; doi:10.12688/f1000research.140107.4)
Supplement: Peer review history

# Reviewer report: Reviewer response for version 3

**DOI:** 10.5256/f1000research.174054.r356660

**Published:** 2025-01-11

**Authors:** Pankaj Roy

**Affiliations:** 1 Central University of Karnataka, Kalaburagi, India

**Critical Review of the Article**: "Investigate the Efficiency of Primary Care Facilities in Emergency Situations by Application of Geographical and Demographic Standards Using GIS"

**1. Title Evaluation: **The title reflects the core focus of the study, emphasising both the geographical and demographic dimensions of primary healthcare facility accessibility in emergency contexts. However, it could be more concise by removing redundant phrasing such as "by application of geographical and demographic standards" and simplifying it to suggested below.

**Suggestion: **One of the best fitted titles may be "Evaluating the Efficiency of Primary Healthcare Facilities in Emergency Situations: A GIS-Based Geographical and Demographic Analysis"

**2. Abstract Review: **The abstract provides a clear overview of the study, including the background, methods, results, and conclusions. However, it lacks specificity regarding the geographical area and demographic standards applied. Including more details about the study area (Sleman District, Indonesia) and the GIS tools utilised would enhance clarity.

**Suggestions:**

1. Specify the key GIS tools and methods in the abstract.
2. Highlight the critical finding that 41 additional primary healthcare centres (PHCs) are required.

**3. Introduction Analysis:**The introduction outlines the importance of primary healthcare in emergencies and introduces GIS as a tool for spatial analysis. However, the rationale behind choosing Sleman District as the study area is poorly elaborated. The introduction would benefit from a more detailed discussion of the region's specific healthcare challenges and the relevance of demographic standards in healthcare planning.

**Suggestions:**

1. Provide a stronger justification for selecting Sleman District.
2. Include more citations to recent studies on GIS applications in healthcare.

**4. Methodology Review: **The methodology section describes the use of ArcGIS 10.5 for analysing the distribution of PHC facilities. It also outlines the applications of geographical standards (coverage distance) and demographic standards (population served per PHC). However, the methodology lacks detailed explanations of the GIS network analysis process, and no clear technical roadmap is provided for replicating the study.

**Strengths:**

1. The use of proximity analysis, spatial overlay, and network analysis is appropriate.
2. The study adheres to Indonesian Ministry of Health regulations.

**Weaknesses:**

1. Insufficient detail on data collection and GIS processing steps.
2. Lack of clarity on using the network analysis in QGIS as an open source alternative of Arc GIS 10.5.
3. Lack of population density maps for better visualisation.

**Suggestions:**

1. Include a technical roadmap or flowchart to illustrate the methodology including the GIS analyses.
2. Provide population density maps to complement the spatial analysis.

**5. Results and Analysis:**

 The results highlight that PHC centres cover 79% of the study area, leaving a significant portion underserved. The analysis identifies a need for 41 additional PHCs to meet the demographic standards set by the Ministry of Health. The spatial distribution maps and network analysis effectively demonstrate the gaps in healthcare access.

**Strengths**

1. Clear identification of underserved areas.
2. Effective use of GIS-based maps to visualise healthcare coverage.

**Weaknesses**

1. The results section focuses strongly on geographical coverage without delving deeply into the demographic implications.
2. Limited discussion on the socioeconomic impact of healthcare access disparities.

**Suggestions**

1. Provide more detailed demographic analysis, including the age and gender distribution of underserved populations.
2. Discuss the potential health outcomes resulting from inadequate PHC coverage.

**6. Discussion and Recommendations: **The discussion section explores the implications of the findings, emphasizing the need for more PHC centers in underserved areas. However, the recommendations lack depth and fail to address practical solutions for improving healthcare accessibility.

**Strengths:**

1. The discussion recognises the need for targeted healthcare planning.
2. The recommendations highlight the importance of data-driven decision-making.

**Weaknesses:**

1. Recommendations are too general and lack actionable steps.
2. The discussion does not consider alternative solutions, such as mobile clinics or telemedicine.

**Suggestions:**

1. Provide specific recommendations for policymakers, such as prioritising regions for new PHC centres.
2. Explore alternative healthcare delivery models, like mobile clinics and telehealth services.

**7. References:**

 The references cited are relevant and credible. However, the article would benefit from more recent studies and a broader range of sources, particularly in GIS applications and healthcare planning.

**Strengths:**

1. The references are appropriate and related to the study's focus.
2. The citation style is consistent.

**Weaknesses:**

1. The references list lacks recent studies.
2. Some key studies on healthcare access models are missing.

**Suggestions:**

1. Include more recent references, especially from the last five years.
2. Expand the references to cover healthcare access models and disaster preparedness.
3. Include more references to recent studies on GIS applications in healthcare.

**8. Grammatical and Stylistic Errors:**

 Upon review, several areas in the article could benefit from grammatical and stylistic improvements to enhance readability and clarity. Below are the sampled grammatical errors identified:

**Sentence Length**

 Many sentences are lengthy and could be split for better readability.

**Example:** "The article presents a valuable analysis of healthcare accessibility using GIS, specifically focusing on the spatial distribution of primary healthcare centres and the gaps in service coverage in the Sleman District of Indonesia."

**Suggested Revision:** "The article presents a valuable GIS-based analysis of healthcare accessibility. It specifically focuses on the spatial distribution of primary healthcare centres and the service gaps in the Sleman District of Indonesia."

**9. Passive Voice:**

 The article uses passive voice frequently, which can make the writing less direct.

**Example:** "The study was conducted to find out the sufficient number of primary healthcare (PHC) centres required."

**Suggested Revision:** "The study aimed to determine the number of primary healthcare (PHC) centres needed."

**Redundancy:**

 Some phrases are redundant and can be simplified.

**Example:** "The article claims that all source data are available through relevant government agencies and geoportals."

**Suggested Revision:** "The article states that all source data are available through government agencies and geoportals."

**Word Choice:**

 Certain words and phrases could be more precise or formal.

**Example:** "The citations provided are relevant to the topic, particularly in the use of GIS in healthcare planning."

**Suggested Revision:** "The citations are relevant, particularly GIS applications in healthcare planning."

**Critical Questions for Improvement**

1. How can the study incorporate socioeconomic data to provide a more comprehensive analysis of healthcare access disparities?
2. What alternative healthcare delivery models can be explored to address the shortfall in PHC centres?
3. How can the findings of this study be integrated into national healthcare planning and policy frameworks?

**Overall Evaluation**

 The article presents a valuable analysis of healthcare accessibility using GIS. However, it could be strengthened by addressing the methodological gaps and providing more practical recommendations. The study's relevance to healthcare planning is clear, but it requires more in-depth exploration of demographic and socioeconomic factors to maximize its impact.

**Final Suggestions**

1.
  1. Include a more detailed methodology section.
  2. Provide comprehensive demographic analysis.
  3. Offer specific, actionable recommendations for policymakers.
  4. Explore alternative solutions for improving healthcare access.

By addressing these areas, the article can significantly enhance its contribution to healthcare planning and GIS applications.

Is the work clearly and accurately presented and does it cite the current literature?

Partly

If applicable, is the statistical analysis and its interpretation appropriate?

I cannot comment. A qualified statistician is required.

Are all the source data underlying the results available to ensure full reproducibility?

Yes

Is the study design appropriate and is the work technically sound?

Yes

Are the conclusions drawn adequately supported by the results?

Partly

Are sufficient details of methods and analysis provided to allow replication by others?

Partly

Reviewer Expertise:

Geography, Geoinformatics, Spatial Analysis, Public Health

I confirm that I have read this submission and believe that I have an appropriate level of expertise to confirm that it is of an acceptable scientific standard, however I have significant reservations, as outlined above.

# Response

**Published:** 2025-12-06

**Authors:** Ali Jarghon

**Affiliations:** Public Health Faculty, Airlangga University, surabaya, EastJava, Indonesia

**1.Title Evaluation**

**Reviewer Comment:** The title should be more concise.

**Response:** Thank you for the suggestion. We agree the proposed title is clearer, but since the article is already published under the current title, it cannot be changed now. We will consider this recommendation for future work.

**2.Abstract Review**

**Reviewer Comment: **The abstract lacks details about the study area and GIS tools used.

**Response:** Thank you for this helpful suggestion. We have revised the abstract by specifying the key GIS tools (ArcGIS 10.5 and QGIS) and the main analytical methods applied, including proximity analysis, spatial overlay, and network analysis. We also added the study area, Sleman District, Indonesia, and highlighted the critical finding that 41 additional primary healthcare centers (PHCs) are required to meet both demographic and geographic standards.

**3.Introduction Analysis:**

**viewer Comment: **The rationale for selecting Sleman District is limited, and more recent GIS studies should be cited.

**Response:** Thank you for this helpful suggestion. Sleman District was chosen as the study area because it has a high population density, mixed urban and rural areas, and a high disaster-risk level. Similar studies have also used GIS to analyze healthcare accessibility and planning

**4. Methodology Review:**

**1- Reviewer  Comment:** Include a technical roadmap or flowchart to illustrate the methodology including the GIS analyses.  

 Response: Thank you for the suggestion. I have added a methodology flowchart to clearly present the GIS analysis process.

**2- Reviewer  Comment:** Provide population density maps to complement the spatial analysis.

 Response:

 We thank the reviewer for this helpful suggestion. A population density map has now been added to the Results section to enhance the spatial analysis and support the demographic assessment of PHC service needs.

**5.  Results and Analysis:**

**1-Reviewer  Comment:** Provide more detailed demographic analysis, including the age and gender distribution of underserved populations.

 Response: Thank you for this helpful suggestion.This study had two goals: a geographical goal to measure PHC coverage distance and area, and a demographic goal to identify the total population served. We did not include age or gender details because this requires field surveys and was outside our scope.

**2-  Reviewer Comment: **Discuss the potential health outcomes resulting from inadequate PHC coverage.

 Response: Thank you for this important suggestion. Inadequate PHC coverage can lead to serious health outcomes, including delayed treatment, increased cases of preventable diseases, and higher maternal and child mortality rates. During disasters or emergencies, limited access to healthcare may also result in greater vulnerability and poorer recovery among affected populations.

**6. Discussion and Recommendations:**

**Reviewer Comment: **The recommendations are too general and need more practical solutions for improving healthcare accessibility.

**Response:** Thank you for this valuable suggestion. 

 I have revised the Discussion and Recommendations section to include specific actions for policymakers and alternative healthcare delivery options.  Policymakers prioritize the establishment of new PHC centers in underserved sub-districts such as Cangkringan, Pakem, Turi, and Prambanan, where service coverage and accessibility are lowest. In addition, I have added recommendations to explore mobile health clinics and telehealth services as complementary solutions to reach remote or disaster-prone communities where building new PHCs may not be immediately feasible. 

**7. References:**

**Reviewer Comment:** References

**Response**: Thank you for this helpful. I have updated the reference.

 8. Grammatical and Stylistic:

  Several areas in the article could benefit from grammatical and stylistic improvements to enhance readability and clarity.

 Response: I appreciate the reviewer’s observation. The manuscript has been carefully revised to improve clarity.

**9. Passive Voice:**

 Reviewer Comment:

 The article uses passive voice frequently, which can make the writing less direct.

 Response:

 We appreciate this suggestion. The manuscript has been carefully revised to reduce the use of passive voice and make sentences more direct and active

# Reviewer report: Reviewer response for version 3

**DOI:** 10.5256/f1000research.174054.r351971

**Published:** 2024-12-28

**Authors:** Hanifa Denny

**Affiliations:** 1 Public Health, Universitas Diponegoro, Semarang, Central Java, Indonesia

Dear authors,

 Thank you for your thorough and thoughtful revisions. I have reviewed your responses and the updated manuscript, and I find that you have sufficiently addressed all the comments and suggestions provided. The additional citations, methodological clarifications, and expanded discussions enhance the manuscript's quality and relevance.

 I have no further suggestions at this time, and I am satisfied with the revisions made.

 Best regards,

 Reviewer

Is the work clearly and accurately presented and does it cite the current literature?

Partly

If applicable, is the statistical analysis and its interpretation appropriate?

Yes

Are all the source data underlying the results available to ensure full reproducibility?

Yes

Is the study design appropriate and is the work technically sound?

Yes

Are the conclusions drawn adequately supported by the results?

Yes

Are sufficient details of methods and analysis provided to allow replication by others?

Yes

Reviewer Expertise:

NA

I confirm that I have read this submission and believe that I have an appropriate level of expertise to confirm that it is of an acceptable scientific standard.

# Reviewer report: Reviewer response for version 2

**DOI:** 10.5256/f1000research.168658.r328429

**Published:** 2024-10-28

**Authors:** Jianwei Li

**Affiliations:** 1 Northwest University, Xi'an, Shaanxi, China

The manuscript, based on demographic data and utilizing GIS spatial network analysis, effectively reveals the spatial alignment between the service coverage of primary healthcare (PHC) centers in the Sleman region of Indonesia and the local population's medical needs. This provides valuable guidance for improving real-world healthcare facilities. However, there are still some shortcomings that need further improvement:

1. The manuscript should clearly explain the GIS network analysis process and include a technical roadmap to help readers better understand the entire data processing procedure.
2. The manuscript should provide a population density map of the study area, enabling readers to better understand the spatial distribution of PHC center services in relation to population density in the region.
3. It is recommended to more comprehensively discuss, in conjunction with the research conclusions, how to improve the PHC service system and propose appropriate research strategies to strengthen the impact of the research conclusions.

Is the work clearly and accurately presented and does it cite the current literature?

Yes

If applicable, is the statistical analysis and its interpretation appropriate?

Partly

Are all the source data underlying the results available to ensure full reproducibility?

Partly

Is the study design appropriate and is the work technically sound?

Partly

Are the conclusions drawn adequately supported by the results?

Partly

Are sufficient details of methods and analysis provided to allow replication by others?

Partly

Reviewer Expertise:

Urban and Regional Planning Studies

I confirm that I have read this submission and believe that I have an appropriate level of expertise to confirm that it is of an acceptable scientific standard, however I have significant reservations, as outlined above.

# Response

**Published:** 2025-12-06

**Authors:** Ali Jarghon

**Affiliations:** Public Health Faculty, Airlangga University, surabaya, EastJava, Indonesia

**1. Reviewer Comment:**

 The manuscript should clearly explain the GIS network analysis process and include a technical roadmap to help readers better understand the entire data processing procedure.

**Response:**

 Thank you for the comment. We have revised the methods to explain the GIS network analysis more clearly and added a small technical roadmap to show the main steps.

**2. Reviewer Comment:**

 The manuscript should provide a population density map of the study area, enabling readers to better understand the spatial distribution of PHC center services in relation to population density in the region

**Response:**

 Thank you for the suggestion. I have added a population density map to the Results section so readers can clearly see the population distribution and how it relates to PHC service coverage.

**3. Reviewer Comment:**

 It is recommended to more comprehensively discuss, in conjunction with the research conclusions, how to improve the PHC service system and propose appropriate research strategies to strengthen the impact of the research conclusions.

**Response:**

 Thank you for the recommendation. I have expanded the discussion to explain more clearly how the PHC service system can be improved based on the study’s findings. I also added simple research strategies to strengthen the impact of the conclusions.

# Reviewer report: Reviewer response for version 2

**DOI:** 10.5256/f1000research.168658.r325253

**Published:** 2024-10-10

**Authors:** Hanifa Denny

**Affiliations:** 1 Public Health, Universitas Diponegoro, Semarang, Central Java, Indonesia

1. Clarity and Accuracy of Presentation and Literature Citations

 The manuscript is generally well-written, and the topic is clearly defined. It focuses on primary healthcare (PHC) center distribution in Sleman District, Indonesia. The research is relevant, especially in public health and healthcare infrastructure planning, and it aligns with current literature on disaster management, GIS application in healthcare, and accessibility standards.

 However, there are instances where the literature citations could be more comprehensive. Some key studies, such as those on healthcare access models or the role of GIS in disaster preparedness, could be expanded. The inclusion of more recent studies on GIS applications in emergency healthcare systems would strengthen the discussion. Reviewers also recommended citing relevant literature, which has been partially addressed but could be further improved by adding these references to reinforce the findings.

 2. Study Design and Academic Merit

 The study's design is appropriate for the research question. By using geographical and demographic standards and GIS, the study provides an insightful approach to assessing PHC center coverage and identifying gaps in service. The integration of GIS-based tools is a strong aspect of the research.

 However, some methodological details, such as the choice of specific geographical and demographic standards, could be justified more explicitly. The reasoning behind the selection of a 5 km radius for service coverage, for example, could be explained with more references to health policy or geographical accessibility standards. Despite this, the study has solid academic merit as it tackles an important issue in public health infrastructure.

 3. Sufficient Details of Methods and Replicability

 The methods section is fairly detailed, especially with the use of GIS and demographic data. The revisions made after peer review have improved the clarity of the methodology, making it easier to follow. However, there are still areas that would benefit from more explicit descriptions. For example: 1). More detailed steps on how the network and proximity analyses were conducted. 2). Clear justification for the use of specific GIS tools and how data layers were integrated.

 Providing these details would enhance the study's replicability by other researchers.

 4. Statistical Analysis and Interpretation

 The study does not heavily rely on complex statistical analysis, as its focus is more on spatial data and accessibility measures. That said, the interpretation of the spatial data is appropriate. The visual representations (maps) of PHC service areas help in understanding the disparities in healthcare coverage.

 One area that could be enhanced is a more in-depth statistical interpretation of the population that remains underserved. While the demographic shortfall is identified, further analysis of its implications on healthcare outcomes would add depth to the interpretation of the results.

 5. Source Data Availability and Reproducibility

 The manuscript claims that all data sources are available, including links to the Indonesian Disaster Management Agency and Sleman District's geoportal. This ensures reproducibility for other researchers wishing to replicate the study. To fulfill this criterion, these links must remain accessible. The manuscript also lists detailed instructions on how to access the relevant datasets.

 6. Conclusions Supported by Results

 The study's conclusions are largely supported by the data presented. The manuscript clearly identifies a significant shortfall in PHC centers in the study area, both geographically and demographically. The need for 41 additional centers is well-justified based on the analyzed data.

 However, a more comprehensive discussion of potential solutions or next steps, such as how local policymakers could address these shortfalls, would strengthen the conclusions' impact. This could include considerations on resource allocation, funding, or the integration of these findings into broader public health planning frameworks.

 Summary of Improvements:

 a. Expand the citation of relevant literature, particularly recent studies on GIS in healthcare and disaster management.

 b. Justify the selection of specific geographical standards (e.g., 5 km radius) more clearly.

 c. Add further details in the methods section to improve replicability.

 d. Enhance the discussion on the implications of the demographic shortfalls and provide more recommendations for future action.

Is the work clearly and accurately presented and does it cite the current literature?

Partly

If applicable, is the statistical analysis and its interpretation appropriate?

Yes

Are all the source data underlying the results available to ensure full reproducibility?

Yes

Is the study design appropriate and is the work technically sound?

Yes

Are the conclusions drawn adequately supported by the results?

Yes

Are sufficient details of methods and analysis provided to allow replication by others?

Yes

Reviewer Expertise:

Public Health

I confirm that I have read this submission and believe that I have an appropriate level of expertise to confirm that it is of an acceptable scientific standard, however I have significant reservations, as outlined above.

# Response

**Published:** 2024-11-06

**Authors:** Ali Jarghon

**Affiliations:** Public Health Faculty, Airlangga University, surabaya, EastJava, Indonesia

Thank you so much for your comments and suggests.

**1. Reviewer comment:**

 Expand the citation of relevant literature, particularly recent studies on GIS in healthcare and disaster management.

**Author comment:** Citation have added upon request.

**2. Reviewer comment:**

 Justify the selection of specific geographical standards (e.g., 5 km radius) more clearly.

**Author comment: **added in Result section, third paragraph (The scope of service within 5 kmThe scope of service within 5 km). The 5 km radius for healthcare service coverage is chosen to enhance accessibility and equity, ensuring patients can reach healthcare within a manageable distance, especially in rural and semi-urban areas. (Regulation of the Minister of Health of the Republic of Indonesia Number 43 Year 2019 Concerning Public Health Centers, 2019). 

**3. Reviewer comment:**

 Add further details in the methods section to improve replicability.

**Author comment: **Added in method section in red font, **Data collection, processing, and analysis** **, **basically Spatial Overlay and Network Analysis.

****

**4. Reviewer comment:**

 Enhance the discussion on the implications of the demographic shortfalls and provide more recommendations for future action.

**Author comment: ** comment upon request, which is now added in the sixth paragraph and highlighted in red font. Additionally, the recommendations have been incorporated as requested.

****

# Reviewer report: Reviewer response for version 2

**DOI:** 10.5256/f1000research.168658.r299981

**Published:** 2024-07-24

**Authors:** Ayed Taran

**Affiliations:** 1 Al al-Bayt University, Al-Mafraq, Jordan

No or only minor changes are required. This means that the aims and research methods are adequate; results are presented accurately; and the conclusions are justified and supported by the data or supporting material.

Is the work clearly and accurately presented and does it cite the current literature?

Partly

If applicable, is the statistical analysis and its interpretation appropriate?

Partly

Are all the source data underlying the results available to ensure full reproducibility?

Yes

Is the study design appropriate and is the work technically sound?

Partly

Are the conclusions drawn adequately supported by the results?

No

Are sufficient details of methods and analysis provided to allow replication by others?

No

Reviewer Expertise:

His research interests include transport services planning and management, the socio-economic impacts of transportation congestion, and urban planning applications, transportation geography, Transport models integrated in the GIS environment for accessibility and management of port systems, GIS and Remote Sensing, Transportation Planning and urban sustainable planning, transport services planning.

I confirm that I have read this submission and believe that I have an appropriate level of expertise to confirm that it is of an acceptable scientific standard.

# Reviewer report: Reviewer response for version 1

**DOI:** 10.5256/f1000research.153436.r253431

**Published:** 2024-06-27

**Authors:** Ayed Taran

**Affiliations:** 1 Al al-Bayt University, Al-Mafraq, Jordan

1. The paper addresses geographical and demographic criteria to verify the ability of primary health care facilities to cover the largest area of ​​the study area and accessible service area use GIS, The study found that there is a significant shortage in the number of primary health care centers in the study area compared to the large population, both on the geographical and demographic levels.

 2. The paper deals with a very important topic.

 3. There are technical issues with the arrangement of the paper as regards methodology, there are issues with presentation of data used and the subsequent methods used for the analysis of data. This includes missing methods. The paper also did not provide sufficient analysis that warrant a good discussion of results. The implications of findings are also missing.

 4. I request the authors to cite the following references:

 (i) Taran A (2023 [ref - 1]).  Measuring Accessibility to Health Care Centers in the City of Al-Mafraq Using Geographic Information Systems. ResearchGate. 2023; 19 (1): 43-55

 (ii) Taran A et al. (2023 [ref - 2]).  The Temporal and Spatial Analysis of Corona Pandemic in Jordan using the Geographic Information System: An Applied Geographical Study. Indonesian Journal of Geography. 2023; 55 (1).

 This is because it is relevant to the topic of study and can be benefited from.

 5) All source data underlying the results are available to ensure complete reproducibility.

 Dr. Ayed Taran

 Al al-Bayt University

Is the work clearly and accurately presented and does it cite the current literature?

Partly

If applicable, is the statistical analysis and its interpretation appropriate?

Partly

Are all the source data underlying the results available to ensure full reproducibility?

Yes

Is the study design appropriate and is the work technically sound?

Partly

Are the conclusions drawn adequately supported by the results?

No

Are sufficient details of methods and analysis provided to allow replication by others?

No

Reviewer Expertise:

His research interests include transport services planning and management, the socio-economic impacts of transportation congestion, and urban planning applications, transportation geography, Transport models integrated in the GIS environment for accessibility and management of port systems, GIS and Remote Sensing, Transportation Planning and urban sustainable planning, transport services planning.

I confirm that I have read this submission and believe that I have an appropriate level of expertise to confirm that it is of an acceptable scientific standard, however I have significant reservations, as outlined above.

## References

[1] : Measuring Accessibility to Health Care Centers in the City of Al-Mafraq Using Geographic Information Systems. *ResearchGate* .2023;19(1) : 10.52939/ijg.v19i1.2499 43-55 10.52939/ijg.v19i1.2499

[2] : The Temporal and Spatial Analysis of Corona Pandemic in Jordan using the Geographic Information System: An Applied Geographical Study. *Indonesian Journal of Geography* .2023;55(1) : 10.22146/ijg.73663 10.22146/ijg.73663

# Response

**Published:** 2024-06-29

**Authors:** Ali Jarghon

**Affiliations:** Public Health Faculty, Airlangga University, surabaya, EastJava, Indonesia

Response to Reviewer 

 Thank you for your comments.

 1. Thank you for your insightful comment. We have thoroughly addressed the geographical and demographic criteria to evaluate the coverage of primary healthcare facilities in the study area using GIS. Our study indeed found a significant shortage of primary healthcare centers relative to the large population, both geographically and demographically. 

 2. Thank you for your positive feedback. We are pleased that you recognize the importance of the topic. Ensuring adequate coverage and accessibility of primary healthcare facilities is crucial for public health, and we hope our study contributes valuable insights to this significant issue.

 3. Thank you for your constructive feedback. In response, we have extensively revised the methodology section to address the technical issues and improve the presentation of data and the methods used for analysis. Furthermore, we have expanded the analysis to provide a more thorough discussion of the results and their implications for healthcare accessibility and policy development. 

 4. Thank you for your feedback. We have ensured that all source data underlying the results are available to guarantee complete reproducibility.

# Response

**Published:** 2024-07-22

**Authors:** Ali Jarghon

**Affiliations:** Public Health Faculty, Airlangga University, surabaya, EastJava, Indonesia

**Author response:**

 Thank you for your insightful comments and expertise.
